# Extraction Optimization and Anti-Tumor Activity of Polysaccharides from *Chlamydomonas reinhardtii*

**DOI:** 10.3390/md22080356

**Published:** 2024-08-02

**Authors:** Zhongwen Liang, Lan Xiong, Ying Zang, Zhijuan Tang, Zhenyu Shang, Jingyu Zhang, Zihan Jia, Yanting Huang, Xiaoyu Ye, Hongquan Liu, Mei Li

**Affiliations:** Guangxi Key Laboratory for Polysaccharide Materials and Modifications, School of Marine and Biotechnology, Guangxi University for Nationalities, Nanning 530006, China; liangzhongwen1129@163.com (Z.L.); 18311818967@163.com (L.X.); 15545488961@163.com (Y.Z.); 18278317940@163.com (Z.T.); 13287897117@163.com (Z.S.); 15123795358@163.com (J.Z.); 15034148159@163.com (Z.J.); 18277977174@163.com (Y.H.); 18077561144@163.com (X.Y.)

**Keywords:** *Chlamydomonas reinhardtii* polysaccharides, ultrasonic-assisted extraction, cancer treatment, anti-tumor activity

## Abstract

*Chlamydomonas reinhardtii* polysaccharides (CRPs) are bioactive compounds derived from *C. reinhardtii*, yet their potential in cancer therapy remains largely unexplored. This study optimized the ultrasound-assisted extraction conditions using response surface methodology and proceeded with the isolation and purification of these polysaccharides. The optimal extraction conditions were identified as a sodium hydroxide concentration of 1.5%, ultrasonic power of 200 W, a solid-to-liquid ratio of 1:25 g/mL, an ultrasonic treatment time of 10 min, and a water bath duration of 2.5 h, yielding an actual extraction rate of 5.71 ± 0.001%, which closely aligns with the predicted value of 5.639%. Infrared analysis revealed that CRP-1 and CRP-2 are α-pyranose structures containing furoic acid, while CRP-3 and CRP-4 are β-pyranose structures containing furoic acid. Experimental results demonstrated that all four purified polysaccharides inhibited the proliferation of cervical (HeLa) hepatoma (HepG-2) and colon (HCT-116) cancer cells, with CRP-4 showing the most significant inhibitory effect on colon cancer and cervical cancer, achieving inhibition rates of 60.58 ± 0.88% and 40.44 ± 1.44%, respectively, and significantly reducing the migration of HeLa cells. DAPI staining confirmed that the four purified polysaccharides inhibit cell proliferation and migration by inducing apoptosis in HeLa cells. CRP-1 has the most significant inhibitory effect on the proliferation of liver cancer cells. This study not only elucidates the potential application of *C. reinhardtii* polysaccharides in cancer therapy but also provides a scientific basis for their further development and utilization.

## 1. Introduction

With the increasing incidence of cancer, more attention is being paid to this serious disease. Changes in living conditions and dietary habits have led to a continuous rise in cancer incidence, with an increasing trend among adolescents and young adults [1]. Clinical treatment methods for tumors mainly include surgery, radiotherapy, and chemotherapy. Additionally, efforts are being made to explore new cancer treatment methods, such as tumor vaccines and nano-anti-tumor drugs [2,3,4]. During cancer treatment, patients often endure a painful process, highlighting the urgent need for new treatment methods. In the search for new anti-tumor drugs, natural polysaccharides have been found to possess anti-tumor activity. Natural polysaccharides inhibit tumor proliferation and metastasis by blocking the cell cycle, inducing apoptosis, inhibiting angiogenesis, and modulating host immunity [5,6]. Furthermore, some studies have reported that natural polysaccharides achieve their anti-tumor activity indirectly by modulating the tumor microenvironment (TME) [7]. Jianzhi Zhang et al. discovered that *Chlorella* polysaccharides have inhibitory effects on colon cancer HCT116 and HCT8 cells [8]. Yi Gao et al. found that *Sargassum* polysaccharides effectively inhibit the growth of lung cancer A549 cells, liver cancer HepG2 cells, and mouse melanoma B16 cells while promoting immune cell proliferation and enhancing the expression levels of cytokines IL-6, IL-1β, iNOS, and TNF-α [9]. Jue Tu et al. demonstrated that *Crocus sativus* polysaccharides exhibit anti-tumor activity in S180 sarcoma mouse tissues by converting tumor-associated macrophages (TAMs) from the tumor-promoting M2 phenotype to the anti-tumor M1 phenotype, thus remodeling the tumor microenvironment [10].

Cervical cancer is the most common malignant tumor in women and a major global health issue. Human papillomavirus (HPV) is one of the primary causes of cervical cancer development [11]. HPV screening and vaccination programs are effective strategies for cervical cancer prevention. However, the lack of early screening and treatment methods for cervical cancer in low- and middle-income countries leads to many deaths annually [12]. Surgery, chemotherapy, radiotherapy, and targeted drugs remain the main treatments for cervical cancer [13]. Traditional treatment methods are expensive, have poor efficacy, and cause significant side effects, making the search for new treatment methods crucial. In recent years, microalgal polysaccharides, derived from marine and freshwater microalgae, have gained widespread attention in scientific research due to their unique physiological activities and potential health benefits. These polysaccharides not only exhibit biological activities such as immunomodulation, antiviral, and antibacterial effects but also play unique roles in inhibiting tumor proliferation [14,15,16]. Guowei Ya’s research indicates that *Lentinan* activates caspase-9 and caspase-3, inducing apoptosis in cervical cancer HeLa cells [17]. Jiao Xu et al. found that *Cordyceps cicadae* polysaccharides inhibit tumor proliferation by blocking the S phase of the cell cycle and inducing apoptosis in cervical cancer HeLa cells [18]. Yue Liu’s study shows that *Rosa* polysaccharides inhibit the proliferation and migration of HeLa and SiHa cells by activating the caspase protein family and the reactive oxygen species (ROS)-mediated mitochondrial pathway [19]. Microalgae are widely found in oceans and freshwater, and often serve as food for aquatic animals. They also play an important role in purifying water bodies and repairing aquatic ecosystems [20,21]. There remains a significant gap in the treatment of cervical cancer with microalgal polysaccharides, which have potential development value. However, the low extraction rate and difficulty in isolation and purification of microalgal polysaccharides hinder their activity exploration.

*C. reinhardtii* is rich in polysaccharides, proteins, lipids, and pigments, making it a beneficial algae widely used in feed and nutritional supplements [22]. However, the anti-tumor activity of *C. reinhardtii* polysaccharide is still unclear, and it is urgent to further explore the anti-tumor activity of *C. reinhardtii* polysaccharide. This study optimized the extraction conditions of *C. reinhardtii* polysaccharides using response surface methodology and purified the polysaccharides using a cellulose DEAE-52 anion exchange column. The anti-tumor activity of *C. reinhardtii* polysaccharides was evaluated using cervical cancer HeLa cells, liver cancer HepG-2 cells, and colon cancer HCT-116 cells, providing scientific evidence and technical support for the development and application of *C. reinhardtii*.

## 2. Results

### 2.1. Single-Factor Experiment

**Figure 1:**
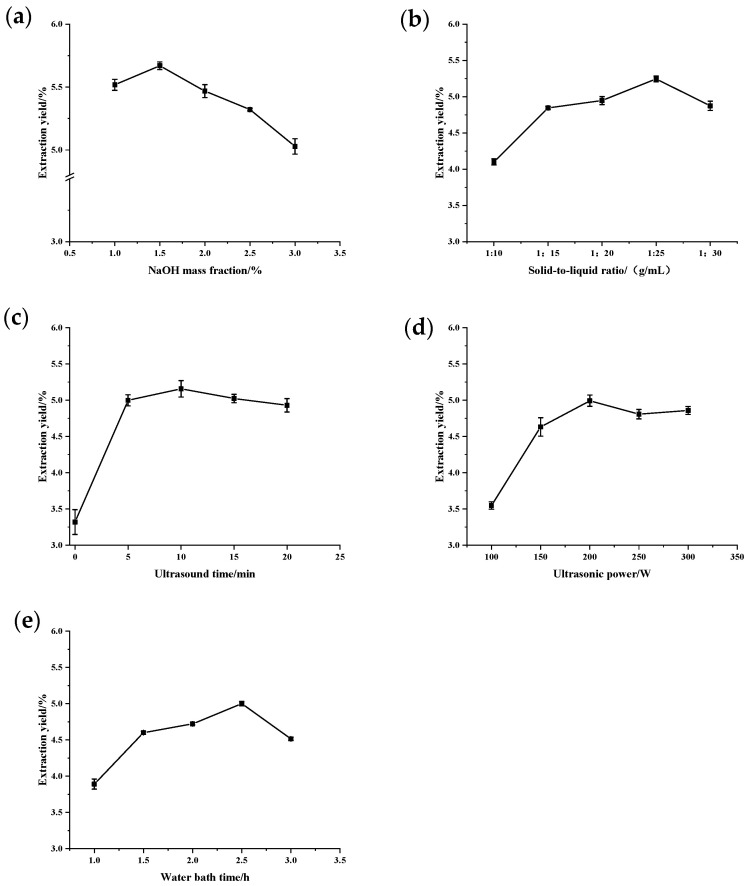
Single-factor test. (**a**) NaOH mass fraction. (**b**) Solid-to-liquid ratio. (**c**) Ultrasound time. (**d**) Ultrasonic power. (**e**) Water bath time. The data are expressed as mean ± standard deviation of at least 3 independent experiments (3 replicates).

As shown in Figure 1a, the polysaccharide extraction rate reached its maximum at a NaOH mass fraction of 1.5%. Beyond this concentration, the extraction rate gradually decreased, likely due to polysaccharide degradation induced by the higher alkaline concentration [23]. Figure 1b demonstrates the effect of the solid-to-liquid ratio on the polysaccharide extraction rate, which first increased and then decreased. The extraction rate reached its peak at a solid-to-liquid ratio of 1:25. Increasing the solid-to-liquid ratio promoted an increase in the solvent volume, thereby increasing the contact area between the solid and liquid phases, which enhanced the ultrasonic wall-breaking effect. However, when the solid-to-liquid ratio exceeded a certain level, all *C. reinhardtii* polysaccharides were dissolved, and further increases in solvent volume did not improve the extraction rate [24].

As shown in Figure 1c, with the increase in ultrasound time, the polysaccharide extraction rate gradually increased. In the range of 0 to 5 min, the polysaccharide extraction rate increased the most, indicating that ultrasound-assisted extraction could significantly increase the polysaccharide extraction rate. When the ultrasound time exceeded 10 min, the polysaccharide extraction rate slowly decreased. This was due to the degradation of polysaccharides when the cells were completely broken [23]. As shown in Figure 1d, the effect of ultrasonic power on the polysaccharide extraction rate of *C. reinhardtii* showed a trend of first increasing and then decreasing. When the ultrasonic power was 200 W, the polysaccharide extraction rate reached its maximum. A further increase in ultrasonic power caused a slow decrease in the polysaccharide extraction rate due to the increased temperature and the degradation of polysaccharides resulting from the increased ultrasonic power [25]. As shown in Figure 1e, with the increase in water bath time, the polysaccharide extraction rate slowly increased. When the water bath time was 2.5 h, the polysaccharide extraction rate reached its maximum. Afterward, the polysaccharide extraction rate began to decrease due to the degradation of polysaccharides caused by long-term high-temperature water bath treatment.

### 2.2. Plackett–Burman Design Analysis

According to the results of the single-factor test, the Plackett–Burman test was designed as shown in Table 1 and Table 2. The results showed that the highest extraction rate of polysaccharide in the second tube was 5.37. The extraction conditions were as follows: sodium hydroxide mass fraction (A) of 1%, solid–liquid ratio (B) of 1:30 g/mL, ultrasonic time (C) of 5 min, ultrasonic power (D) of 250 W, and water bath time (E) of 3 h. The significance and variance analysis of the test results are shown in Table 2. The F value and *p* value of the model are 30.71 and 0.0003, respectively, indicating that the model is meaningful and the results are significant. Among them, the three factors of A, B, and D have a significant effect on the extraction rate of polysaccharides, and the significant effect is B > A > D. Therefore, in order to study the interaction between A, B, and D, the ultrasonic time was fixed for 10 min and the water bath time was 2.5 h.

### 2.3. Response Surface Analysis

The Box–Behnken experimental design and results are shown in Table 3 and Table 4. Based on the response surface experiment results, the software was used to analyze the response values, resulting in the actual factor regression equation for the polysaccharide extraction process:

Y = 1.62A + 0.039725B + 0.2507C − 0.0013AB − 0.021AC + 0.00024BC − 0.26A^2^ − 0.000107B^2^ − 0.0115C^2^ − 7.22375

The variance and significance analysis of the experimental results are shown in Table 4. The regression model *p*-value was 0.0042 (*p* < 0.05), indicating that the model was significant. The lack-of-fit *p*-value was 0.7631 (*p* > 0.05), indicating it was not significant, which means the model adequately fitted the experimental data with low error and minimal influence from unknown factors. The effects of different factors on the polysaccharide extraction rate were C > B > A, which means the effects were in the order of solid-to-liquid ratio > ultrasonic power > sodium hydroxide mass fraction. The model’s coefficient of variation (CV) was 1.94% < 10%, and the signal-to-noise ratio (RSN) was 8.504 > 4.0, indicating that the experimental results had high reliability and relevance.

**Figure 2:**
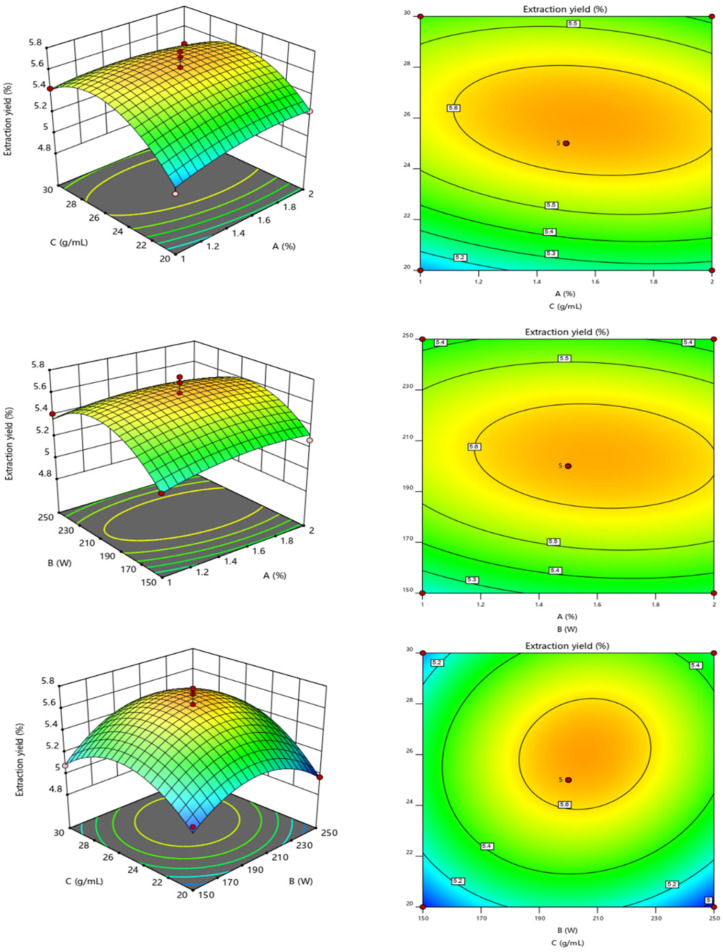
The effects of mass fraction of NaOH, ultrasonic power, and solid–liquid ratio on the extraction rate of polysaccharide were studied. A: mass fraction of NaOH; B: ultrasonic power; C: solid–liquid ratio.

Using Design Expert 12, three-dimensional response surface plots were drawn to clearly reflect the interaction effects of different factors on the polysaccharide extraction rate. The strength of interactions between factors was represented by contour lines. As shown in Figure 2, the interaction between any two of the three factors had a maximum value, with the interaction between the solid-to-liquid ratio and ultrasonic power having the most significant impact on the polysaccharide extraction rate. The software predicted the optimized extraction conditions to be a sodium hydroxide mass fraction of 1.555%, ultrasonic power of 217.309 W, and a solid-to-liquid ratio of 1:25.807 g/mL, resulting in an extraction rate of 5.639%. Based on practical operations, the fixed conditions were a sodium hydroxide mass fraction of 1.5%, ultrasonic power of 200 W, a solid-to-liquid ratio of 1:25 g/mL, ultrasound time of 10 min, and water bath time of 2.5 h, resulting in an extraction rate of 5.71 ± 0.001%. This result was close to the predicted value, indicating that the model could accurately predict and simulate the extraction rate and optimal extraction conditions for intracellular polysaccharides of *C. reinhardtii*.

### 2.4. Isolation and Purification of C. reinhardtii Polysaccharides

**Figure 3:**
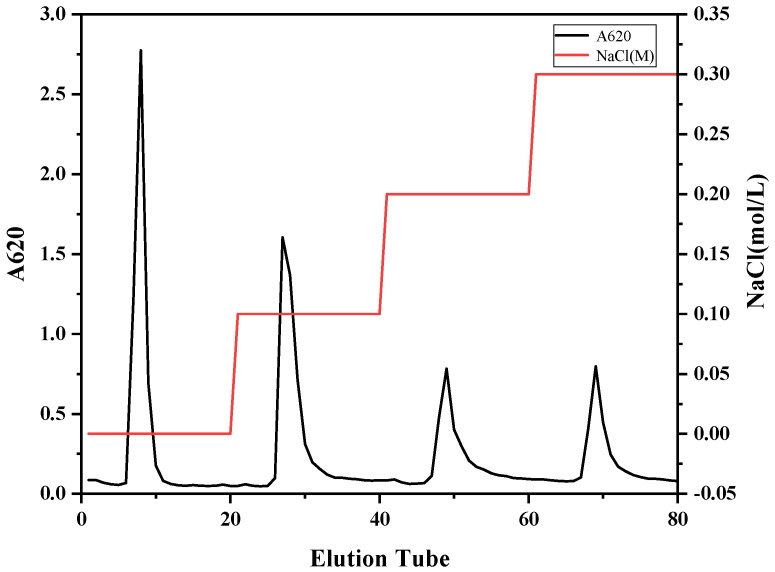
The elution profile of crude polysaccharide from *C. reinhardtii* on DEAE-cellulose anion exchange chromatography column. The black curve represents the elution curve of polysaccharides measured by the anthrone sulfuric acid method. The red curve represents different concentrations of NaCl.

Cellulose DEAE52 depends on the charge difference of different components. By adjusting the ionic strength of the eluent, the separation of different charged components was achieved. CRP was purified by cellulose DEAE-52 anion exchange chromatography column. As shown in Figure 3, four obvious elution peaks were obtained, named CRP-1 (eluted with ultrapure water), CRP-2 (eluted with 0.1 M NaCl), CRP-3 (eluted with 0.2 M NaCl) and CRP-4 (eluted with 0.3 M NaCl). The content of CRP-1 was the highest, and the content of CRP-2, CRP-3 and CRP-4 decreased gradually.

### 2.5. UV Spectroscopy Analysis

**Figure 4:**
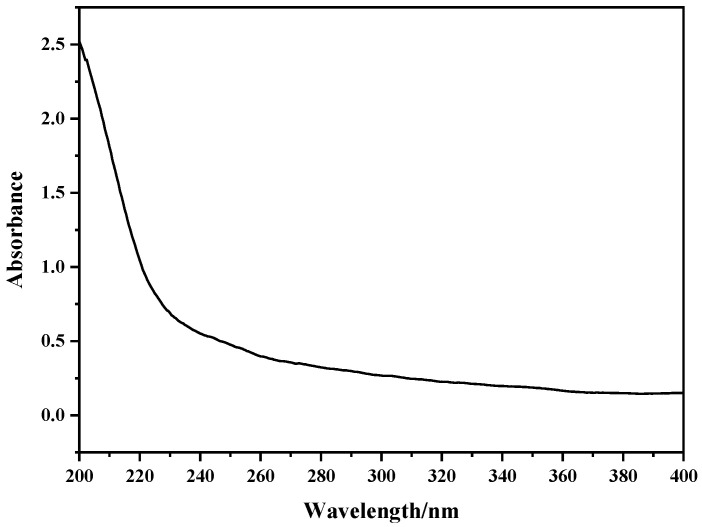
Ultraviolet spectra of *C. reinhardtii* polysaccharides. There is no absorption peak at 260 nm or 280 nm.

As shown in Figure 4, there was no obvious absorption peak at 260 nm and 280 nm, indicating that the protein in the sample was successfully removed after trichloroacetic acid treatment, and the crude polysaccharide of *C. reinhardtii* contained almost no nucleic acid and protein.

### 2.6. Infrared Spectroscopy Analysis

**Figure 5:**
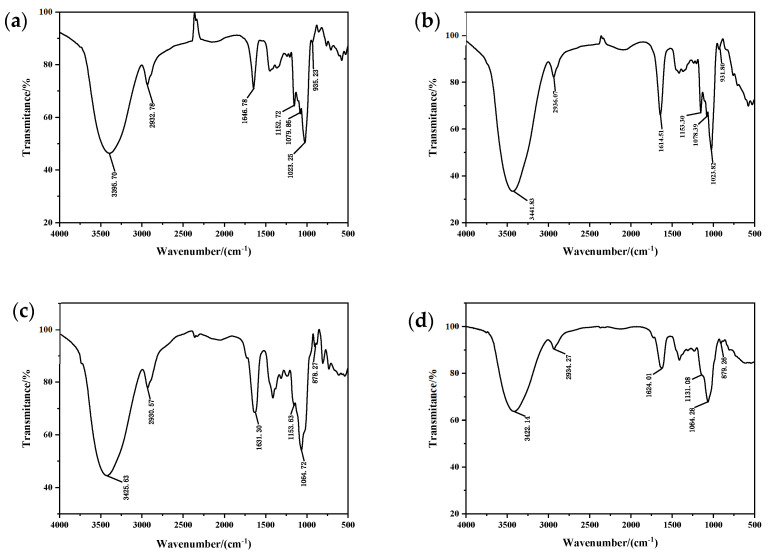
Infrared spectra of four purified polysaccharides. (**a**) CRP-1. (**b**) CRP-2. (**c**) CRP-3. (**d**) CRP-4.

The FT-IR spectra of *C. reinhardtii* polysaccharides, shown in Figure 5, indicated the characteristic stretching vibration peaks of -OH at 3395–3450 cm^−1^ and the stretching vibration peaks due to C-H at around 2930 cm^−1^ [26]. The absorption peaks observed at 1400–1200 cm^−1^ were attributed to the bending vibrations of C-H, which are characteristic peaks of carbohydrates [27,28]. The peak near 1630 cm^−1^ represented the characteristic stretching vibration peak of the C=O in furoic acid [29]. The absorption peaks at 1200–1000 cm^−1^ were caused by the stretching vibrations of C-O-C, C-O-H, and C-O on the pyran ring [16]. The peaks at 870.32 and 879.26 cm^−1^ were the characteristic absorption peaks of α-glycosidic bonds, while the peaks at 935.23 and 931.80 cm^−1^ were the characteristic absorption peaks of β-glycosidic bonds [30]. In summary, CRP-1 and CRP-2 were identified as α-pyranose containing furoic acid, while CRP-3 and CRP-4 were β-pyranose containing furoic acid.

### 2.7. Toxic Effects of CRP on Vero Cells

**Figure 6:**
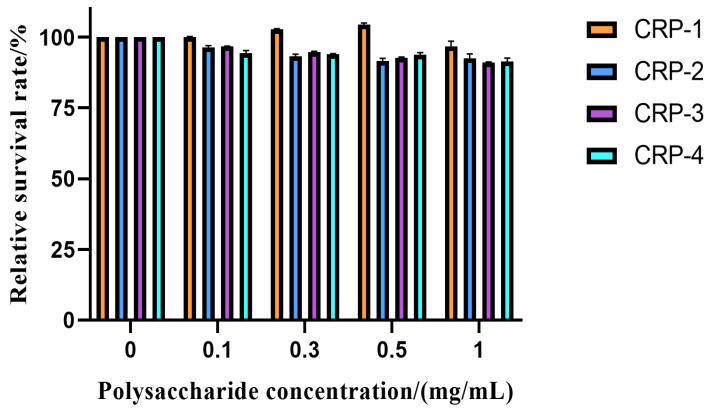
Toxic effect of *C. reinhardtii* polysaccharide on Vero cells. There was no cytotoxicity to Vero cells after treatment with CRP-1, CRP-2, CRP-3 and CRP-4 for 24 h. The data are expressed as mean ± standard deviation of at least 3 independent experiments (3 replicates).

Drugs selectively inhibit cancer cells and have low cytotoxicity to normal cells. It is a principle that must be adhered to in the process of finding new anti-tumor drugs [31]. Vero cells are derived from African green monkey kidney cells and play a huge role in the production of viruses and vaccines. Vero cells are considered to be a normal cell line and can be used to evaluate cytotoxicity [31]. Margaret Selina Modimola et al. used Vero cells to evaluate the cytotoxicity of bacterial secondary metabolites [32]. The cytotoxicity of *C. reinhardtii* polysaccharides on Vero cells is shown in Figure 6. The four purified polysaccharides did not inhibit Vero cells at concentrations below 1 mg/mL, with a relative cell viability greater than 90%. The relative cell viability of CRP-1 gradually increased with increasing concentration, showing a certain proliferation-promoting effect. Therefore, 1 mg/mL was chosen as the working concentration for subsequent anti-tumor experiments.

### 2.8. Effect of CRPs on Tumor Cell Proliferation

**Figure 7:**
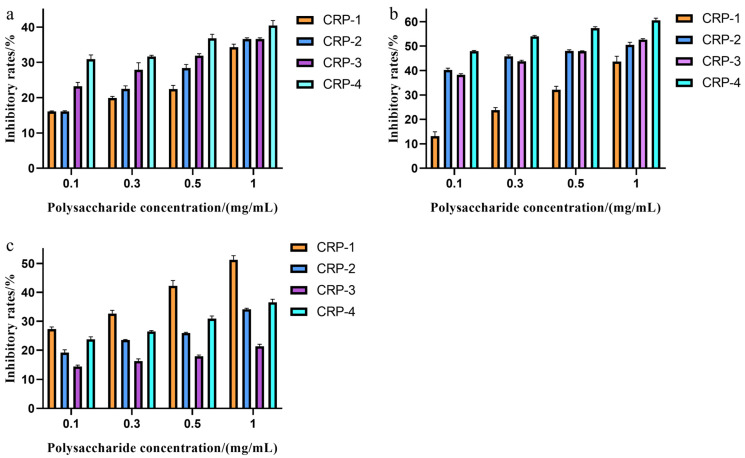
Effects of *C. reinhardtii* polysaccharide on the proliferation of different tumor cells. HCT-116, HeLa, and HepG2 cells were treated with four purified polysaccharides for 24 h, and the proliferation of tumor cells was inhibited. (**a**) HCT-116 cells. (**b**) HeLa cells. (**c**) HepG-2 cells. The data are expressed as mean ± standard deviation of at least 3 independent experiments (3 replicates).

**Figure 8:**
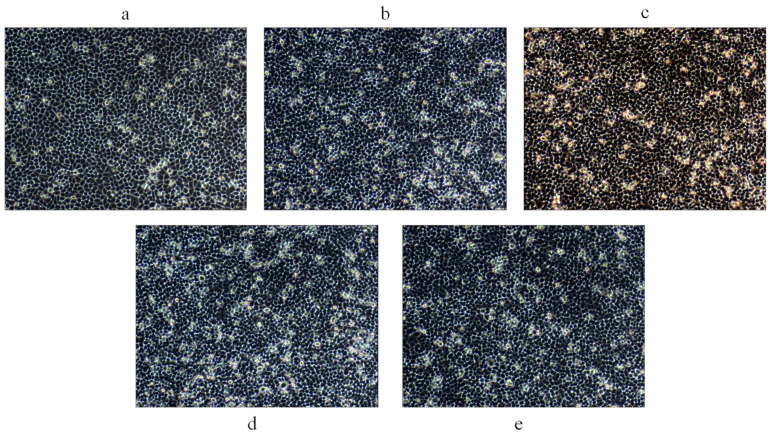
The effect of CRP-4 on the morphology of cervical cancer HeLa cells. (**a**) Control; (**b**) 0.1 mg/mL CRP-4; (**c**) 0.3 mg/mL CRP-4; (**d**) 0.5 mg/mL CRP-4; (**e**) 1 mg/mL CRP-4. After HeLa cells were treated with CRP-4 for 24 h, HeLa cells showed different morphological changes compared with the control group, including morphological abnormalities, increased cell gap, cell shrinkage, blurred contour, and darkening of luster. Microscope magnification is 200×.

An MTT assay was used to detect the effect of four purified polysaccharides on cell proliferation. The effect of CRPs on colon cancer HCT-116 cells is shown in Figure 7a. All four purified polysaccharides had some inhibitory effect on the proliferation of HCT-116 cells, with the inhibition rate increasing with concentration at non-cytotoxic concentrations. The maximum inhibition rates of CRP-1, CRP-2, CRP-3, and CRP-4 on colon cancer HCT-116 cells were 34.31 ± 0.83%, 36.65 ± 0.31%, 36.65 ± 0.31%, and 40.44 ± 1.44%, respectively, with CRP-4 showing the highest inhibition rate. The effect of CRPs on cervical cancer HeLa cells is shown in Figure 7b. At non-cytotoxic concentrations, all four purified polysaccharides inhibited the proliferation of HeLa cells, with the inhibition rate proportional to concentration. At 1 mg/mL, the inhibition rates of CRP-1, CRP-2, CRP-3, and CRP-4 on cervical cancer HeLa cells were 43.68 ± 2.15%, 50.49 ± 1.04%, 52.61 ± 0.44%, and 60.58 ± 0.88%, respectively, with IC_50_ values of 1.425 mg/mL, 0.825 mg/mL, 0.7033 mg/mL, and 0.1433 mg/mL, respectively. CRP-4 showed the highest inhibition rate on cervical cancer HeLa cells, demonstrating significant anti-tumor activity. Comparatively, CRP exhibited the highest inhibition rate on cervical cancer HeLa cells. The morphological changes of cervical cancer HeLa cells are shown in Figure 8. Compared to the control group, CRP-4 treatment led to cell morphological changes, including cell shrinkage, abnormal shape, and reduced intercellular connections. The effect of CRP on hepatoma HepG-2 cells is shown in Figure 7c. The four purified polysaccharides had inhibitory effects on the proliferation of HepG-2 cells, and the inhibition rate increased with the increase in concentration at non-cytotoxic concentrations. The maximum inhibition rates of CRP-1, CRP-2, CRP-3 and CRP-4 on HepG-2 cells were 51.23 ± 1.44%, 34.18 ± 0.32%, 21.35 ± 0.72%, and 36.5 ± 1.12%, respectively, and the inhibition rate of CRP-1 was the highest. The results showed that the four purified polysaccharides could inhibit the proliferation of colon cancer (HCT-116), cervical cancer (HeLa), and liver cancer (HepG-2) cells.

### 2.9. Cell Scratch Assay

**Figure 9:**
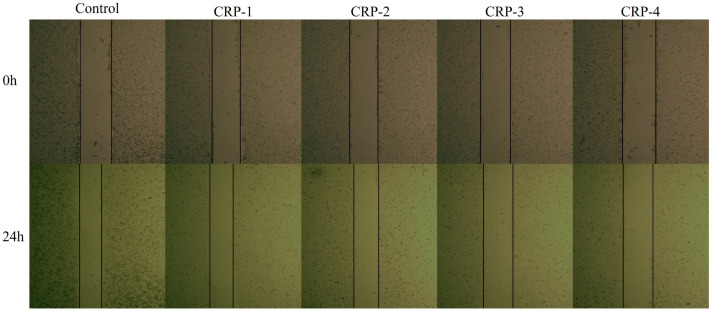
Effect of *C. reinhardtii* polysaccharide on migration of cervical cancer HeLa cells. Compared with the control group, the migration ability of HeLa cells was inhibited after treatment with four purified polysaccharides for 24 h. Microscope magnification is 100×.

**Figure 10:**
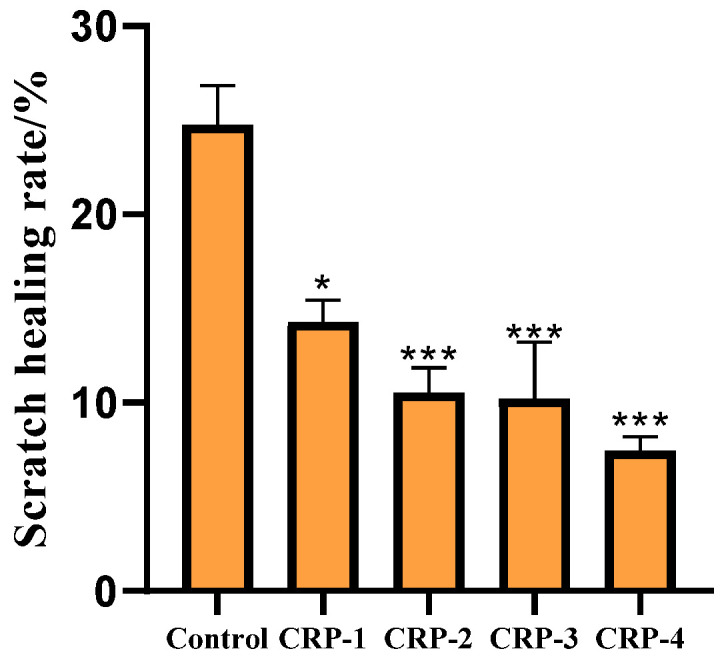
The effects of four purified polysaccharides on the migration of cervical cancer HeLa cells. The scratches were quantitatively analyzed by Image J 1.8 software and similar results were obtained from two other independent experiments. The data are expressed as mean ± standard deviation of at least 3 independent experiments (3 replicates). An asterisk (*) indicates a significant difference (*p* < 0.05), while three asterisks (***) indicate a highly significant difference (*p* < 0.01).

The cell migration assay is shown in Figure 9 and Figure 10. Untreated HeLa cells exhibited significant migration, with the scratch area noticeably reduced after 24 h. Compared to the control group, HeLa cells treated with CRP-1, CRP-2, CRP-3, and CRP-4 showed significantly reduced migration ability. All four purified polysaccharides inhibited the migration of cervical cancer HeLa cells, with CRP-4 showing the most significant inhibition of HeLa cell migration.

### 2.10. DAPI Staining

**Figure 11:**
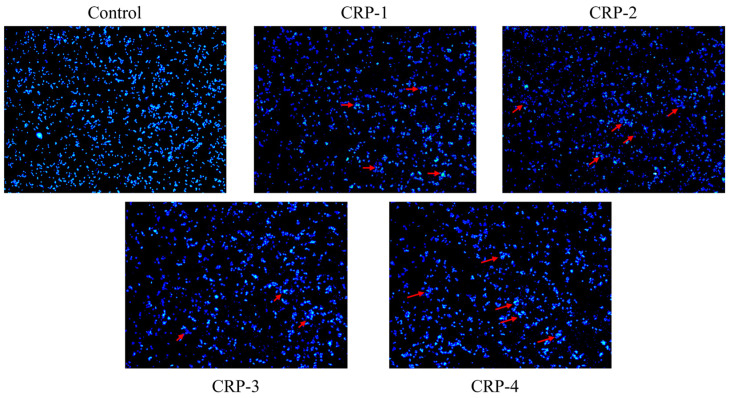
DAPI staining was used to observe the effect of *C. reinhardtii* polysaccharide on the apoptosis morphology of cervical cancer HeLa cells. Red arrows represent chromatin condensation, nuclear fragmentation, formation of apoptotic bodies, bright blue aggregates or dots.

DAPI is commonly used for live cell staining. To evaluate whether CRPs’ inhibitory effect on HeLa cell growth was related to apoptosis, both the control group and the CRP-treated groups were stained with DAPI and observed under a fluorescence microscope. The result of DAPI staining is shown in Figure 11. After treatment with CRP-1, CRP-2, CRP-3, and CRP-4, significant nuclear changes were observed, such as chromatin condensation, nuclear fragmentation, and the formation of apoptotic bodies, which appeared as bright blue aggregates or dots, indicative of apoptosis [33]. In contrast, the nuclei of the control group showed a uniform blue color, with even chromatin and smooth edges, indicating intact nuclei. This suggested that CRPs induce apoptosis in cervical cancer HeLa cells.

## 3. Discussion

Polysaccharides are important active components in microalgae, exhibiting antioxidant, anti-tumor, immune-enhancing, anti-inflammatory, and lipid-lowering activities, thus showing great potential in treating various diseases [34,35]. The side effects of drugs causing adverse reactions during treatment highlight the urgent need for a safe and effective therapeutic method. *C. reinhardtii*, as a nutritional supplement and health food, has unclear polysaccharide bioactivity. This study extracted *C. reinhardtii* polysaccharides using ultrasound-assisted extraction, isolated and purified them with a cellulose DEAE-52 anion exchange column, and explored their in vitro anti-tumor activities.

Low extraction rates and difficulties in isolation and purification are major obstacles to the development and application of natural polysaccharides. Current extraction methods for natural polysaccharides include hot water extraction, acid–alkaline extraction, microwave-assisted extraction, ultrasound-assisted extraction, and enzymatic extraction [36,37]. Different extraction techniques have potential impacts on the extraction rate, structure, and bioactivity of polysaccharides, making the extraction process crucial in polysaccharide research. The Box–Behnken design (BBD) in response surface methodology (RSM) uses statistical and mathematical methods to optimize parameters and the process between the response surfaces. As an effective statistical method, it has been widely used to evaluate the plant polysaccharide extraction process, including ultrasound-assisted extraction [24]. This study combined hot water extraction, alkaline extraction, and ultrasound-assisted extraction to extract *C. reinhardtii* polysaccharides and optimized the extraction conditions through RSM. The software predicted the optimal extraction conditions to be a sodium hydroxide mass fraction of 1.555%, ultrasonic power of 217.309 W, and a solid-to-liquid ratio of 1:25.807 g/mL, resulting in an extraction rate of 5.639%. Based on practical operations, the fixed conditions were a sodium hydroxide mass fraction of 1.5%, ultrasonic power of 200 W, solid-to-liquid ratio of 1:25 g/mL, ultrasound time of 10 min, and water bath time of 2.5 h, yielding an extraction rate of 5.71 ± 0.001%, which was close to the predicted value. The polysaccharides were isolated and purified using a cellulose DEAE-52 anion exchange column, resulting in four different fractions. Infrared spectroscopy provided preliminary structural characterization, indicating that CRP-1 and CRP-2 are α-pyranoses containing furoic acid, while CRP-3 and CRP-4 are β-pyranoses containing furoic acid.

Cancer is a widely concerned disease mainly due to its high mortality rate and poor prognosis. Many studies have shown that natural polysaccharides (including those from plants and microorganisms) possess anti-tumor activities. For instance, *Spirulina* polysaccharides significantly inhibit the proliferation of lung cancer A549 cells, and *Ganoderma* polysaccharides inhibit the proliferation of breast cancer MCF7 cells by promoting apoptosis [38,39]. In this study, CRP-1, CRP-2, CRP-3 and CRP-4 inhibited the proliferation of colon cancer HCT-116 cells, liver cancer HepG-2 cells, and cervical cancer HeLa cells at non-cytotoxic concentrations, and the effect on cervical cancer HeLa cells was the most significant. Interestingly, CRP-1 had low anti-tumor activity against cervical cancer HeLa cells, but had the highest anti-tumor activity against liver cancer HepG-2 cells. This may be due to the different modes of action of CRP-1 on different tumor cells, resulting in differences in anti-tumor activity of CRP-1 on different tumor cells. The inhibition rates of the four purified polysaccharides on HeLa cells were 43.68 ± 2.15%, 50.49 ± 1.04%, 52.61 ± 0.44%, and 60.58 ± 0.88%, with IC_50_ values of 1.425 mg/mL, 0.825 mg/mL, 0.7033 mg/mL, and 0.1433 mg/mL, respectively. CRP-4 exhibited the highest inhibition rate on cervical cancer HeLa cells, and all four purified polysaccharides significantly inhibited HeLa cell migration. DAPI staining indicated that the four purified polysaccharides activated anti-tumor activity by inducing apoptosis in HeLa cells. Dong Zhen’s study showed that *Ganoderma* polysaccharides induce apoptosis in MCF-7 cells by activating caspase-3 and caspase-9 [40]. Reem Al Monla et al. demonstrated that brown algae polysaccharides induced apoptosis in colon cancer HCT-116 cells, confirmed by DAPI staining and flow cytometry [41]. Our DAPI staining results were similar to their findings, although we did not further verify the results using flow cytometry. Natural polysaccharides are mainly composed of β-glycosidic bonds. Compared with α-glycosidic bonds, polysaccharides with β-glycosidic bonds show higher anti-tumor activity [42]. Our study further suggests that CRP-3 and CRP-4 are composed of β-glycosidic bonds, and their anti-tumor activity is higher than that of CRP-1 and CRP-2.

In summary, our results indicated that *C. reinhardtii* polysaccharides have good anti-tumor activity and hold promising prospects for cervical cancer treatment. However, this study has certain limitations, such as not elucidating the anti-tumor mechanisms of *C. reinhardtii* polysaccharides and lacking animal experimental verification. Additionally, further structural characterization of *C. reinhardtii* polysaccharides was not performed, providing direction and a theoretical basis for our future research.

## 4. Materials and Methods

### 4.1. Materials and Chemicals

Chlamydomonas reinhardtii and Vero, HCT-116, HepG-2, and HeLa cells were preserved in our laboratory. MTT and DEAE-cellulose (DEAE-52) were purchased from Beijing Solarbio Science & Technology Co., Ltd. (Beijing, China). FBS, DMEM, and PBS used for cell culture were all purchased from Biological Industries Co., Ltd. (Kibbutz Beit Haemek, Israel).

### 4.2. Extraction of Intracellular Polysaccharides from C. reinhardtii

One gram of accurately weighed *C. reinhardtii* powder was mixed with NaOH solution in a certain ratio. The mixture was treated with an ultrasonic disruptor to release intracellular polysaccharides, followed by a water bath at 60 °C. After the water bath, the mixture was centrifuged at 8000 r/min for 10 min to collect the supernatant, to which four volumes of anhydrous ethanol were added and left to stand overnight. The precipitate was collected by centrifugation the next day, redissolved, and treated with 3% trichloroacetic acid solution to remove proteins, then left to stand overnight. The supernatant was collected by centrifugation the next day, precipitated again with ethanol, and the precipitate was freeze-dried to obtain *C. reinhardtii* intracellular polysaccharides.

### 4.3. Determination of Polysaccharide Content

The polysaccharide content of *C. reinhardtii* was determined using the anthrone–sulfuric acid method. First, a glucose standard curve was prepared by diluting a 1 mg/mL glucose stock solution. Polysaccharide solution and 0.1% anthrone–sulfuric acid solution were mixed in a 1:3 ratio, shaken thoroughly, and incubated in a 100 °C water bath for 10 min. After cooling to room temperature, the absorbance at 620 nm was measured [43,44]. The glucose standard curve was plotted, with the equation Y = 0.0029X + 0.0801 and R^2^ = 0.9996. For the polysaccharide solution, 100 μL was mixed with 300 μL of 0.1% anthrone–sulfuric acid solution, shaken thoroughly, and incubated in a 100 °C water bath for 10 min. After cooling to room temperature, the absorbance at 620 nm was measured to calculate the polysaccharide extraction rate.

### 4.4. Single-Factor Experiment

The extraction conditions for *C. reinhardtii* polysaccharides were fixed as follows: NaOH mass fraction (A) of 2%, ultrasonic power (B) of 200 W, ultrasound time (C) of 10 min, solid-to-liquid ratio (D) of 1:20 g/mL, and water bath time (E) of 2 h. By changing one factor while keeping the others constant, the effects of the five factors on the polysaccharide extraction rate were investigated. The experimental design is shown in Table 5.

### 4.5. Plackett–Burman Experimental Design

The PB experiment is a method designed to screen for significant influencing factors based on single-factor experiment results. The effects of the NaOH mass fraction (A), solid-to-liquid ratio (B), ultrasound time (C), ultrasonic power (D), and water bath time (E) on the polysaccharide extraction rate were studied. The conditions with the highest extraction rates from the single-factor experiments were chosen as the high level (1) and low level (−1) for the PB experiment, designed using Design-Expert 12 software. The design of the 12 groups of experiments and their polysaccharide extraction rates are shown in Table 6.

### 4.6. Response Surface Design

Based on the results of the single-factor and PB experiments, a Box–Behnken experiment was designed. The NaOH mass fraction (A), ultrasonic power (B), and solid-to-liquid ratio (C) were chosen as variables, and the polysaccharide extraction rate was the response value. The experimental design is shown in Table 7.

### 4.7. Isolation and Purification of Polysaccharides

CRPs were isolated and purified using a cellulose DEAE-52 anion exchange column. A 100 mg CRP sample was dissolved in ultrapure water and filtered through a 0.22 μm Millipore filter. Sequential elution was performed with 0, 0.1, 0.2, and 0.3 mol/L NaCl solutions, collecting 9 mL per fraction at a flow rate of 3 mL/min. The polysaccharide content was detected using the anthrone–sulfuric acid method. Four fractions were collected, dialyzed, and freeze-dried, then labeled CRP-1, CRP-2, CRP-3, and CRP-4 according to their elution order.

### 4.8. Characterization of Intracellular Polysaccharides from C. reinhardtii

A 0.1% polysaccharide solution was prepared and scanned using a UV-Vis spectrophotometer (Cary60, Agilent, California, USA) within the range of 200–400 nm to confirm the absence of proteins and nucleic acids [45]. CRP-1, CRP-2, CRP-3, and CRP-4 were analyzed using the potassium bromide (KBr) pellet method. Each sample was mixed with KBr in a 1:100 ratio, dried, and ground thoroughly in an agate mortar to form thin pellets. Fourier Transform Infrared Spectroscopy (FT-IR) was performed on a MAGNA-IR 550 spectrometer (Thermo Fisher, Massachusetts, USA) in the range of 4000–500 cm^−1^ to acquire the infrared spectra of the samples [46].

### 4.9. Cell Culture

HeLa, HepG-2, HCT-116, and Vero cells were cultured in DMEM medium containing 10% fetal bovine serum and 1% penicillin–streptomycin (100 units/mL) at 37 °C in a humidified atmosphere with 5% CO_2_.

### 4.10. Cytotoxicity Assay

The cytotoxicity of the polysaccharides was measured using the MTT assay [47,48]. Vero cells were seeded in 96-well plates at a density of 1 × 10^5^ cells/mL and cultured overnight until 90% confluence. The old culture medium was removed, and the cells were washed once with PBS. Then, 200 μL of polysaccharide solutions (0.1, 0.3, 0.5, 1 mg/mL of CRP-1, CRP-2, CRP-3, CRP-4) was added. The blank group received 200 μL of complete medium. Each group had six replicates. After 24 h of incubation, 20 μL of MTT was added, and the plates were incubated at 37 °C for 4 h. The culture medium was removed, and 150 μL of DMSO was added, shaken for 10 min to dissolve the formazan crystals, and the absorbance at 490 nm was measured using a microplate reader (BioTek, Epoch, Vermont, USA). Cell viability was calculated based on the absorbance values, with As representing the absorbance of the experimental groups and Ac representing that of the control groups.

$$Cell\;viability=\frac{As}{Ac}\times 100\%$$

### 4.11. In Vitro Anti-Tumor Activity

HCT-116, HepG-2, and HeLa cells were seeded in 96-well plates at a density of 1 × 10^5^ cells/mL and cultured overnight until 90% confluence. The old culture medium was removed, and the cells were washed once with PBS. Then, 200 μL of polysaccharide solutions (0.1, 0.3, 0.5, 1 mg/mL of CRP-1, CRP-2, CRP-3, CRP-4) was added. The blank group received 200 μL of complete medium. Each group had six replicates. After 24 h of incubation, 20 μL of MTT was added, and the plates were incubated at 37 °C for 4 h. The culture medium was removed, and 150 μL of DMSO was added; the solution was shaken for 10 min to dissolve the formazan crystals, and the absorbance at 490 nm was measured. Inhibitory rates were calculated based on the absorbance values, where As represents the absorbance of the experimental groups and Ac represents the absorbance of the control groups.

$$Inhibitory\;rates=1-\frac{As}{Ac}\times 100\%$$

### 4.12. Cell Migration Assay

Cell migration was assessed using a scratch test. HeLa cells were seeded in 6-well plates at a density of 1.5 × 10^5^ cells/mL and incubated for 24 h until confluent. A pipette tip was used to scratch the cell monolayer, and 2 mL of 0.1 mg/mL polysaccharide solutions (CRP-1, CRP-2, CRP-3, CRP-4) was added. After 24 h, the scratch area was observed under an inverted fluorescence microscope (CKX41-A32PH-FL, Olympus, Tokyo, Japan). The scratch healing rate was calculated according to the migration area. S_0_ and S_24_ represent the scratch area at 0 and 24 h after scratch formation.

$$Scratch\;healing\;rate=1-\frac{S_{24}}{S_{0}}\times 100\%$$

### 4.13. DAPI Staining

DAPI staining was used to observe changes in nuclear morphology [49,50]. HeLa cells were seeded in 6-well plates at a density of 1.5 × 10^5^ cells/mL and incubated for 24 h until confluent. Then, 2 mL of 1 mg/mL polysaccharide solutions (CRP-1, CRP-2, CRP-3, CRP-4) was added. After 24 h, the culture medium was removed, and the cells were washed once with PBS. The cells were fixed in 4% paraformaldehyde for 10 min, washed with PBS 1–2 times, stained with 400 μL DAPI staining solution for 5 min per well, and washed twice with PBS. Fluorescence was observed using a fluorescence microscope (DM2000 LE, Leica, Wetzlar, Germany).

## 5. Conclusions

The optimal extraction conditions for *C. reinhardtii* polysaccharides were a sodium hydroxide mass fraction of 1.5%, ultrasonic power of 200 W, solid-to-liquid ratio of 1:25 g/mL, ultrasound time of 10 min, and water bath time of 2.5 h, resulting in an extraction rate of 5.71 ± 0.001%, which was close to the predicted value. Infrared analysis revealed that CRP-1 and CRP-2 were α-pyranoses containing furoic acid, while CRP-3 and CRP-4 were β-pyranoses containing furoic acid. All four purified polysaccharides inhibited the proliferation of colon cancer HCT-116 cells, liver cancer HepG-2 cells, and cervical cancer HeLa cells, with the most significant effect on cervical cancer. DAPI staining demonstrated that *C. reinhardtii* polysaccharides inhibited the proliferation and migration of cervical cancer HeLa cells by inducing apoptosis. However, the molecular mechanisms underlying the anti-tumor effects of *C. reinhardtii* polysaccharides require further investigation and validation. The findings of this study provide a foundation for the development and application of the bioactivity of *C. reinhardtii* polysaccharides and offer new insights into cancer treatment.

## Figures and Tables

**Table 1:** Plackett–Burman experimental design and results.

Run Order	A/%	B/(g/mL_)	C/min	D/W	E/h	Extraction Yield/%
1	−1	1	1	1	−1	5.26
2	−1	1	−1	1	1	5.37
3	−1	1	1	−1	1	5.27
4	1	−1	−1	−1	1	4.5
5	1	1	−1	1	1	5
6	1	1	1	−1	−1	5.25
7	−1	−1	1	−1	1	4.77
8	1	1	−1	−1	−1	5.1
9	−1	−1	−1	1	−1	4.45
10	1	−1	1	1	−1	4.23
11	1	−1	1	1	1	4.29
12	−1	−1	−1	−1	−1	4.8

**Table 2:** Plackett–Burman test results and variance analysis.

Source	Sum of Squares	df	Mean Square	F-Value	*p*-Value	Significance
Model	1.78	5	0.3558	30.71	0.0003	***
A	0.2002	1	0.2002	17.28	0.006	***
B	1.48	1	1.48	127.48	0.0001	***
C	0.0019	1	0.0019	0.1618	0.7014	
D	0.099	1	0.099	8.55	0.0265	*
E	0.001	1	0.001	0.087	0.7779	
Residual	0.0695	6	0.0116			
Cor Total	1.85	11				

An asterisk (*) indicates a significant difference (*p* < 0.05), while three asterisks (***) indicate a highly significant difference (*p* < 0.01).

**Table 3:** Box–Behnken experimental design and results.

Run Order	A/%	B/W	C/(g/mL)	Extraction Yield/%
1	2	150	25	5.27
2	1.5	250	30	5.25
3	1.5	150	20	5.04
4	1.5	150	30	5.08
5	1	250	25	5.41
6	1	200	30	5.43
7	1.5	200	25	5.57
8	1	200	20	5.04
9	1.5	250	20	4.97
10	1.5	200	25	5.78
11	2	200	30	5.43
12	1	150	25	5.2
13	2	250	25	5.35
14	1.5	200	25	5.73
15	2	200	20	5.25
16	1.5	200	25	5.64
17	1.5	200	25	5.48

**Table 4:** Box–Behnken experimental variance analysis.

Source	Sum of Squares	df	Mean Square	F-Value	*p*-Value	Significance
Model	0.8779	9	0.0975	9.04	0.0042	***
A	0.0061	1	0.0061	0.5607	0.4784	
B	0.019	1	0.019	1.76	0.226	
C	0.099	1	0.099	9.18	0.0191	*
AB	0.0042	1	0.0042	0.3916	0.5513	
AC	0.011	1	0.011	1.02	0.3457	
BC	0.0144	1	0.0144	1.33	0.2859	
A2	0.0178	1	0.0178	1.65	0.24	
B2	0.3013	1	0.3013	27.92	0.0011	***
C2	0.348	1	0.348	32.26	0.0080	***
Residual	0.755	7	0.0108			
Lack of fit	0.0173	3	0.0058	0.3969	0.7631	
Pure Error	0.0582	4	0.0145			
Cor Total	0.9534	16				

An asterisk (*) indicates a significant difference (*p* < 0.05), while three asterisks (***) indicate a highly significant difference (*p* < 0.01).

**Table 5:** Single-factor experiment condition setting.

Factor	Condition Setting				
A (%)	1	1.5	2	2.5	3
B (W)	100	150	200	250	300
C (min)	0	5	10	15	20
D (g/mL)	1:15	1:20	1:25	1:30	1:35
E (h)	1	1.5	2	2.5	3

**Table 6:** Plackett–Burman experimental factors and levels.

Level	Factor				
	A (%)	B (g/mL)	C (min)	D (W)	E (h)
−1	1	1:20	5	150	2
1	2	1:30	15	250	3

**Table 7:** Response surface test factors and levels.

Level	Factor		
	A: NaOH Mass Fraction/%	B: Ultrasonic Power/W	C: Solid-to-Liquid Ratio/(g/mL)
−1	1	150	20
0	1.5	200	25
1	2	250	30

## Data Availability

The data presented in this study are available on request from the corresponding author.
